# SRA Suppresses Antiviral Innate Immune Response in Macrophages by Limiting TBK1 K63 Ubiquitination via Deubiquitinase USP15

**DOI:** 10.1128/spectrum.02028-22

**Published:** 2022-11-07

**Authors:** Lei Li, Jialiang Luo, Zhengyumeng Zhu, Qishan Xu, Ping Wang, Bo Chang, Di Wang, Lu Yu, Xiao Lu, Jia Zhou, Daming Zuo, Qingyun Chen

**Affiliations:** a Department of Medical Laboratory, Guangdong Province Key Laboratory of Immune Regulation and Immunotherapy, School of Laboratory Medicine and Biotechnology, Southern Medical University, Guangzhou, Guangdong, P.R. China; b Department of Immunology, School of Basic Medical Sciences, Southern Medical University, Guangzhou, Guangdong, P.R. China; c Medical Research Institute, Guangdong Provincial People’s Hospital, Guangdong Academy of Medical Sciences, Guangzhou, Guangdong, P.R. China; d Department of Dermatology, Dermatology Hospital of Southern Medical University, Southern Medical University, Guangzhou, Guangdong, P.R. China; e Microbiome Medicine Center, Department of Laboratory Medicine, Zhujiang Hospital, Southern Medical University, Guangzhou, Guangdong, P.R. China; University of Florida

**Keywords:** innate antiviral response, scavenger receptor A, TANK-binding kinase 1, ubiquitination

## Abstract

The innate immune system is the first line of host defense against microbial infections. During virus infection, pattern recognition receptors (PRRs) are engaged to detect specific viral components, such as viral RNA or DNA, and regulate the innate immune response in the infected cells or immune cells. Our previous study demonstrated that scavenger receptor A (SRA), an important innate PRR, impaired the anti-hepatitis B virus (HBV) response in hepatocytes. Given that SRA is primarily expressed in macrophages, here, we assessed the function of SRA expressed in macrophages in response to RNA or DNA viral infection. SRA-deficient (SRA^−/−^) mice showed reduced susceptibility to viral infection caused by vesicular stomatitis virus (VSV) or herpes simplex virus 1 (HSV-1). In the virus-infected SRA^−/−^ mice, compared with their wild-type (WT) counterparts, we observed low amounts of virus accompanied by enhanced interferon (IFN) production. Furthermore, SRA significantly inhibited the phosphorylation of TANK-binding kinase 1 (TBK1) and interferon regulatory factor 3 (IRF3). We provided biochemical evidence showing that SRA directly interacts with the N-terminal kinase domain (KD) of TBK1, resulting in the limitation of its K63-linked ubiquitination. Moreover, we demonstrated that SRA negatively regulates the activity of TBK1 by promoting the recruitment of ubiquitin-specific protease 15 (USP15) to deubiquitinate TBK1. In summary, we have identified the connection between SRA and the TBK1/IRF3 signaling pathway in macrophages, indicating a critical role of SRA in the regulation of host antiviral immunity.

**IMPORTANCE** During virus infection, PRRs are engaged to detect specific viral components, such as viral RNA or DNA, and regulate the innate immune response in the infected cells or other immune cells. We reported that deficiency of SRA, an important innate PRR, promoted IRF3 activation, type I IFN production, and innate antiviral responses against RNA and DNA viruses *in vivo* and *in vitro*. Furthermore, the biochemical analysis showed that SRA directly interacts with the KD domain of TBK1 and limits its K63-linked polyubiquitination, reducing TBK1 activation. Further analyses determined that SRA is a modulator for TBK1 activation via the recruitment of USP15, which delineated a previously unrecognized function for SRA in innate antiviral immunity.

## INTRODUCTION

Innate immunity is the first line of defense against pathogens that invade the host. In response to virus infection, which depends on the detection of pathogen-associated molecular patterns (PAMPs), innate immune cells such as macrophages and dendritic cells (DCs) are activated by germ line-encoded pattern recognition receptors (PRRs), including Toll-like receptor 3 (TLR3) (1), retinoic acid-inducible gene I (RIG-I) (2–4), melanoma differentiation-associated protein 5 (MDA5) (5), and cyclic GMP-AMP synthase (cGAS) (6). Upon recognition of the invading viruses, the PRRs recruit downstream adaptors, including TIR domain-containing adapter-inducing interferon-β (TRIF), mitochondrial antiviral-signaling protein (MAVS), and stimulator of interferon genes (STING), which activate the downstream kinases TANK-binding kinase 1 (TBK1) and inhibitor of nuclear factor-kappa B kinase epsilon (IKKε). Activated TBK1 induces the production of type I interferon (IFN-I) and proinflammatory cytokines by activating interferon regulatory factor 3/7 (IRF 3/7) and nuclear factor-kappa B (NF-κB) (7). The activation of TBK1 is regulated in various ways, such as protein ubiquitination and phosphorylation. The ubiquitination of TBK1 is critical for the phosphorylation of IRF3 and IRF7 to activate downstream signaling pathways. Ubiquitin is usually attached to TBK1 as K48-, K63-, and K33-linked chain, depending on the specific conditions (8–10). The particular TBK1 ubiquitination way is critical for producing IFN-I or inflammatory cytokines (10). Families of deubiquitinating enzymes (DUBs) cleave specialized ubiquitin linkages, thus switching the ubiquitin-dependent processes (11). A few members of DUBs cleaving K63-linked ubiquitin chains on TBK1 have been shown to afflict the IFN-I antiviral response (12). Meanwhile, many components relating to the interaction between TBK1 and DUBs were also determined, consequently regulating the IFN-I signaling.

Scavenger receptor A (SRA) (also called CD204) is expressed mainly in myeloid cells and is known as a phagocytic receptor recognizing a series of ligands early on (13). SRA plays a critical role in safeguarding against invading pathogen infections and maintaining immune balance through acting as an innate PRR that can recognize and clear invaders or modify the self-molecules (14). Notably, emerging studies pointed out that SRA participates in the pathogenesis of inflammatory diseases and that its immunomodulatory function may be uncoupled from its endocytic/phagocytic activity. For the innate immune response, SRA might also act as an adaptor molecule or coreceptor with other PRRs, including TLRs, nucleotide oligomerization domain (NOD)-like receptors (NLRs), RIG-I, and MDA5 (15–18). Our previous study showed that SRA regulates the ubiquitination of adaptor proteins in the innate immune signaling pathway (16, 19).

This study examined RNA and DNA viral replication and IFN response in wild-type (WT) and SRA-deficient (SRA^−/−^) mice. Our data suggested that SRA deficiency promoted vesicular stomatitis virus (VSV) and herpes simplex virus 1 (HSV-1) viral clearance *in vivo*. Furthermore, we provided evidence confirming that SRA impairs the VSV and the HSV-1-induced IFN signaling pathway activation by decreasing K63-linked ubiquitination of TBK1, consequently reducing the production of IFN-I and resulting in persistent viral infection.

## RESULTS

### Virus infection upregulates SRA expression in macrophages.

**FIG 1:**
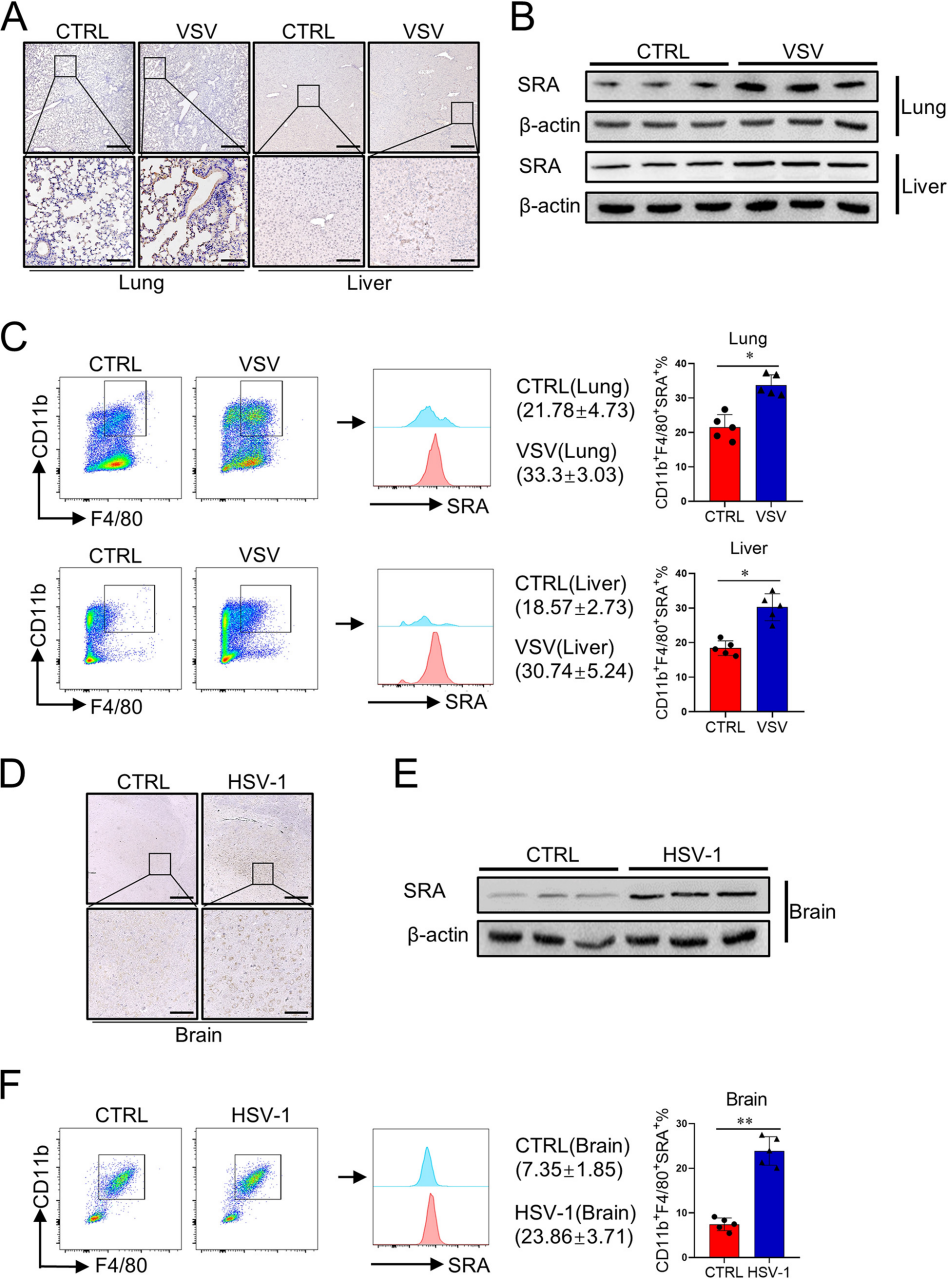
The expression of SRA is upregulated after virus infection. WT mice (*n* = 5 mice/group) were hydrodynamically injected with VSV (2.5 × 10^7^ PFU/mouse) through the tail vein. Mice were sacrificed after a 24-h virus infection. (A) The expression level of SRA in mice lungs and liver was assessed by immunohistochemical staining. (B, C) The level of SRA on MNCs from the lung and liver was analyzed by Western blotting (B) and flow cytometry (C). WT mice (*n* = 5 mice/group) were inoculated with HSV-1 (2 × 10^6^ PFU/eye) on both eyes. Mice were sacrificed after a 5-day virus infection. (D) The expression level of SRA in mice brains was assessed by immunohistochemical staining. (E, F) The level of SRA on MNCs from the brain was analyzed by Western blotting (E) and flow cytometry (F). Scale bars = 500 μm (top) and 100 μm (bottom). *, *P* < 0.05; **, *P* < 0.01; determined by unpaired Student’s *t*-tests. Data are representative of three independent experiments with similar results.

We first evaluated SRA expression in mice infected with either RNA virus VSV or DNA virus HSV-1. WT mice were injected with VSV (2.5 × 10^7^ PFU/mouse) for 24 h through the tail vein, and histology analysis and immunoblotting assay were then performed. Immunohistochemical staining indicated that the expression level of SRA in the lung and liver was higher after VSV infection (Fig. 1A). Considering that SRA is mainly expressed in myeloid cells (20), tissue mononuclear cells (MNCs) were isolated for subsequent analysis. The level of SRA in MNCs increased significantly in both lung and liver tissues from a mouse model of VSV infection (Fig. 1B). Flow cytometry analysis revealed that SRA expression strongly increased on CD11b^+^ F4/80^+^ macrophages (Fig. 1C). Additionally, we assessed brain SRA expression in the mouse model of HSV-1 infection. Immunohistochemical staining showed that the level of SRA in the brain was highly elevated in mice infected by HSV-1 (Fig. 1D). Similarly, the level of SRA in brain MNCs increased significantly in HSV-1-infected mice (Fig. 1E). Fluorescence-activated cell sorter (FACS) analysis also showed that SRA expression strongly increased in CD11b^+^ F4/80^+^ macrophages from the brain tissue (Fig. 1F). Together, these data demonstrated that virus infection significantly induces SRA expression in tissue macrophages.

### SRA deficiency results in decreased susceptibility of mice to viral infection.

**FIG 2:**
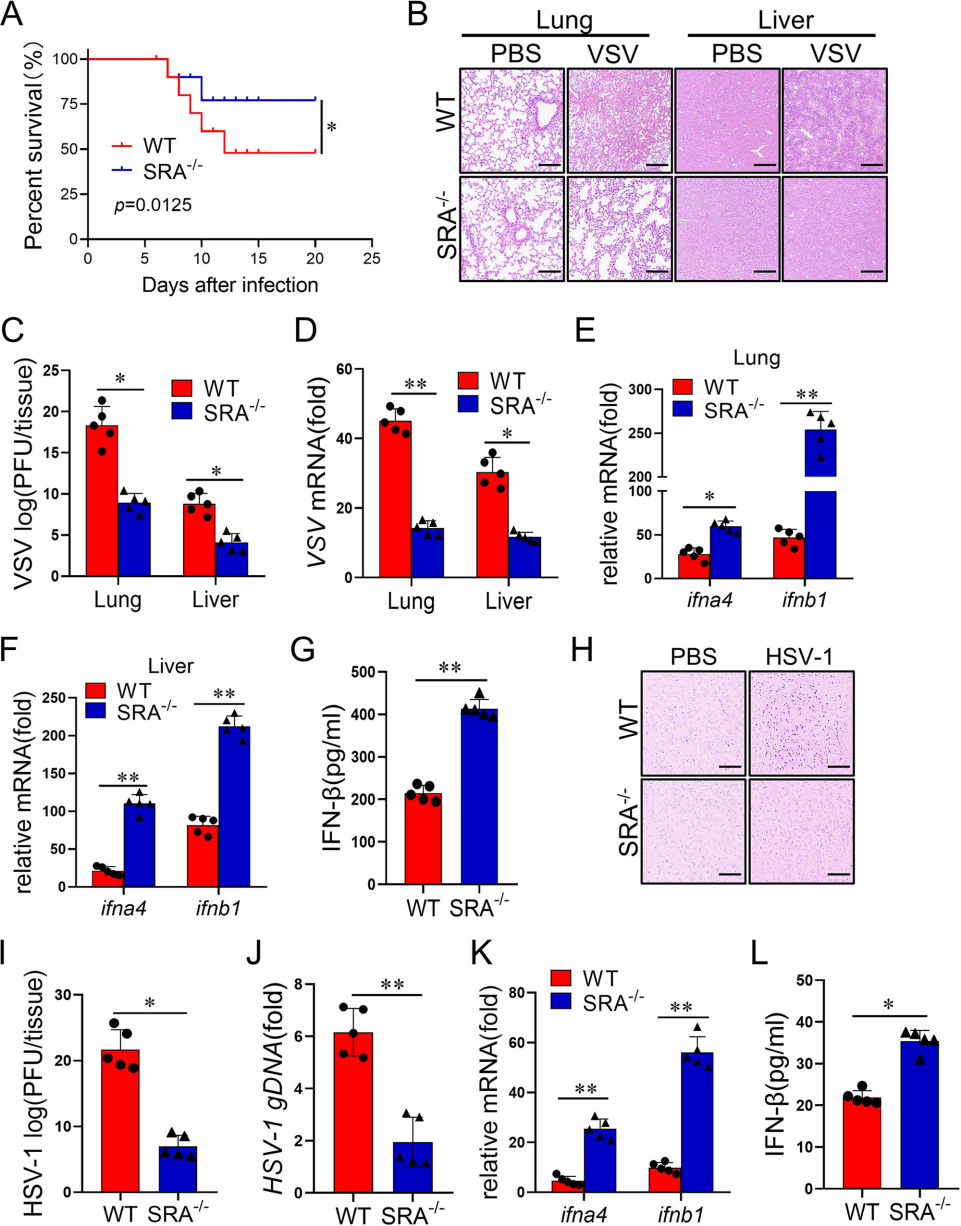
SRA ablation has reduced susceptibility to viral infection. WT and SRA^−/−^ mice (*n* = 5 mice/group) were hydrodynamically injected with VSV (1 × 10^8^ PFU/mouse) through the tail vein. (A) The survival of WT and SRA^−/−^ mice was monitored within 20 days after infection. (B) Representative images of H&E staining in the lung and liver section on 24 h infection. (C, D) The evaluation of viral concentration was assessed by viral titer (C) and VSV mRNA (D). (E, F) The mRNA levels of ifna4 and ifnb1 in the lung (E) and liver (F) after 24 h infection. (G) Serum levels of IFN-β were determined by ELISA. WT and SRA^−/−^ mice (*n* = 5 mice/group) were inoculated with HSV-1 (2 × 10^6^ PFU/eye) on both eyes for 5 days. (H) Representative images of H&E staining in the lung and liver section on 24 h infection. (I, J) The evaluation of viral concentration was assessed by viral titer (I) and HSV-1 genomic DNA (J). (K) The mRNA levels of ifna4 and ifnb1 in mice brains. (L) Serum levels of IFN-β were determined by ELISA. Scale bars = 100 μm. ns, not significant; *, *P* < 0.05; **, *P* < 0.01; determined by unpaired Student’s *t*-tests. Data are representative of three independent experiments with similar results.

To define the effect of SRA on virus infection in mice, WT and SRA^−/−^ mice were injected with VSV (1 × 10^8^ PFU/mouse) via the tail vein, and the survival rate of mice was recorded. The result showed that SRA^−/−^ mice, compared with their WT littermates, showed improved survival after the VSV challenge (Fig. 2A). Compared with the WT mice, VSV-infected SRA^−/−^ mice displayed low inflammatory cell infiltration and attenuated pathological changes in the lung and liver (Fig. 2B). The VSV titers and VSV-G transcript levels in lung and liver were significantly lower in virus-infected SRA^−/−^ mice than in the WT control mice (Fig. 2C and 2D). IFN-I has a critical role in the immune response to viral infection (21). Our data revealed that, upon VSV administration, mRNA expression of type I IFNs (e.g., ifna4 and ifnb1) was significantly increased in SRA^−/−^ mice compared with that in WT mice (Fig. 2E and F). Accordingly, attenuated tissue damage in SRA^−/−^ mice was accompanied by increased secretion of IFN-β (Fig. 2G). Next, we evaluated whether SRA plays a potential role in anti-DNA virus infections, such as HSV-1. We observed attenuated pathological changes and less inflammatory cell infiltration in the brain tissues from HSV-1-infected SRA^−/−^ mice compared with those from WT counterparts (Fig. 2H). Furthermore, there were lower HSV-1 titers and HSV-1 genomic DNA (gDNA) copies in the brain of SRA^−/−^ mice than in WT controls upon the HSV-1 challenge (Fig. 2I and 2J). Moreover, brain mRNA expression of IFNA4 and IFNB1 was significantly enhanced in SRA^−/−^ mice compared with that in WT mice after HSV-1 administration (Fig. 2K). Accordingly, the SRA^−/−^ mice produced more IFN-β in peripheral blood than WT mice after HSV-1 infection (Fig. 2L). These findings show that SRA deficiency limits RNA virus and DNA virus infection *in vivo*.

### SRA impairs antiviral effect and IFN-I production in macrophages.

**FIG 3:**
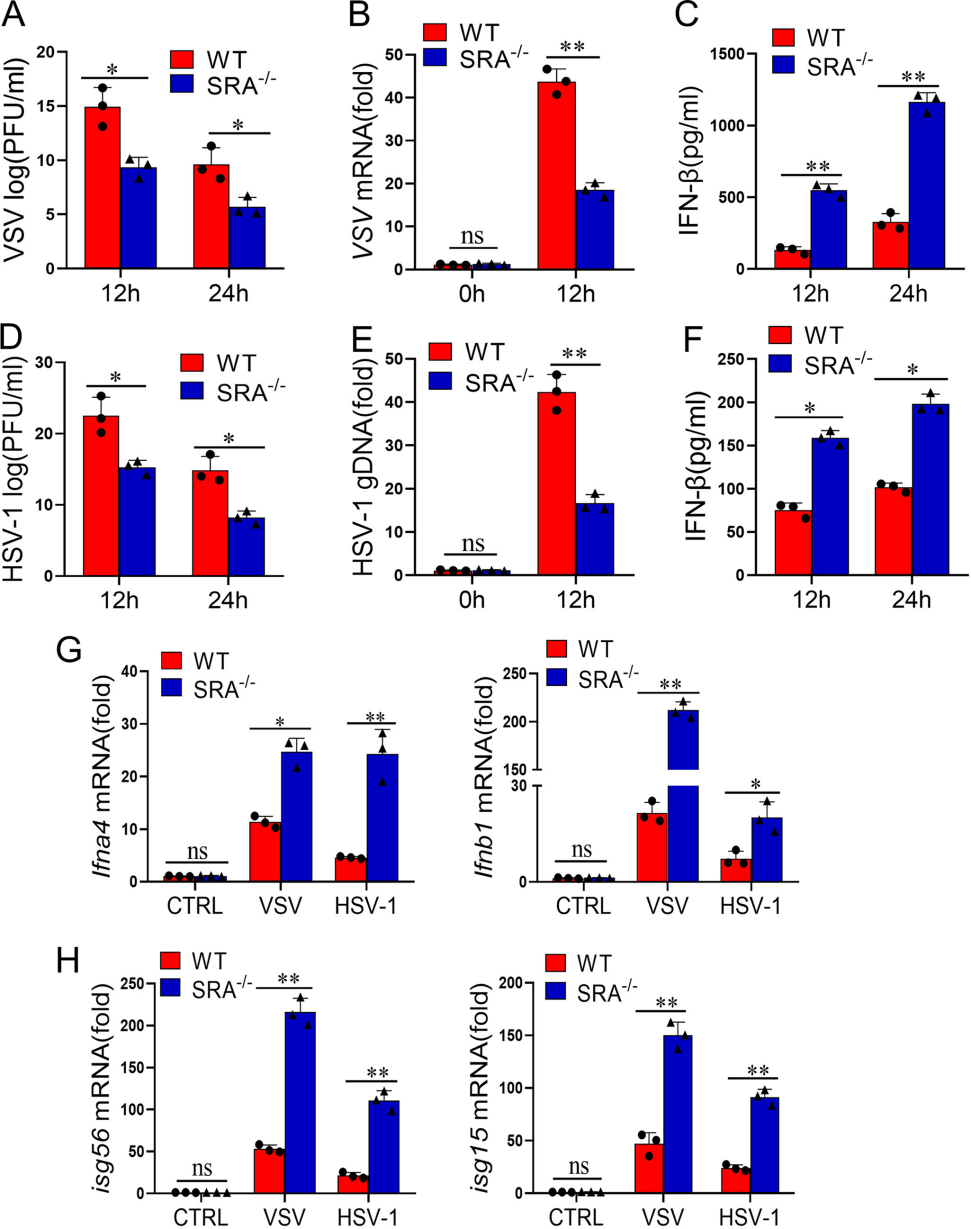
SRA deficiency enhances antiviral immune response in macrophages. (A to C) BMDMs from WT and SRA^−/−^ mice were infected with VSV (MOI = 0.1). The evaluation of viral concentration was assessed by viral titer (A) and VSV mRNA (B). The levels of IFN-β from cell supernatants were determined by ELISA (C). BMDMs from WT and SRA^−/−^ mice were infected with HSV-1 (MOI = 10). (D to F) The evaluation of viral concentration was assessed by viral titer (D) and HSV-1 genomic DNA (E). The levels of IFN-β from cell supernatants were determined by ELISA (F). (G and H) The mRNA levels of ifna4, ifnb1 (G), isg56, and isg15 (H) in BMDMs infected with VSV or HSV-1 for 24 h. ns, not significant; *, *P* < 0.05; **, *P* < 0.01; determined by unpaired Student’s *t*-tests. Data are representative of three independent experiments with similar results.

To further validate the function of SRA in viral infection, we assessed the antiviral function of SRA in macrophages, which are at the first line of host defense and can produce great amounts of IFN-I. Bone marrow-derived macrophages (BMDMs) from WT and SRA^−/−^ mice were infected with VSV (multiplicity of infection [MOI] = 0.1). SRA^−/−^ macrophages demonstrated lower VSV titers (Fig. 3A) and VSV-G transcript levels (Fig. 3B) than WT macrophages after VSV stimulation. Similarly, the secretion of IFN-β from VSV-infected SRA^−/−^ macrophages was higher than in WT macrophage controls (Fig. 3C). Consistently, we found reduced HSV-1 titers and HSV-1 gDNA in SRA^−/−^ macrophages compared with WT macrophages infected with HSV-1 (MOI = 10) (Fig. 3D and 3E). Furthermore, we evaluated the production of IFN-β from virus-stimulated macrophages. The secretion of IFN-β from HSV-1-infected SRA^−/−^ macrophages was higher than that of WT controls (Fig. 3F). Additionally, in response to virus stimulation, the mRNA expression levels of ifna4 and ifnb1 significantly increased in SRA^−/−^ macrophages compared with those in WT macrophages (Fig. 3G). Moreover, SRA deficiency sharply increased the mRNA production of ISG15 and ISG56 in virus-stimulated macrophages (Fig. 3H). Overall, these results indicated that the absence of SRA enhances IFN-I production and immune suppression of viral infection in macrophages.

### SRA negatively regulates the TBK1/IRF3 signaling pathway.

**FIG 4:**
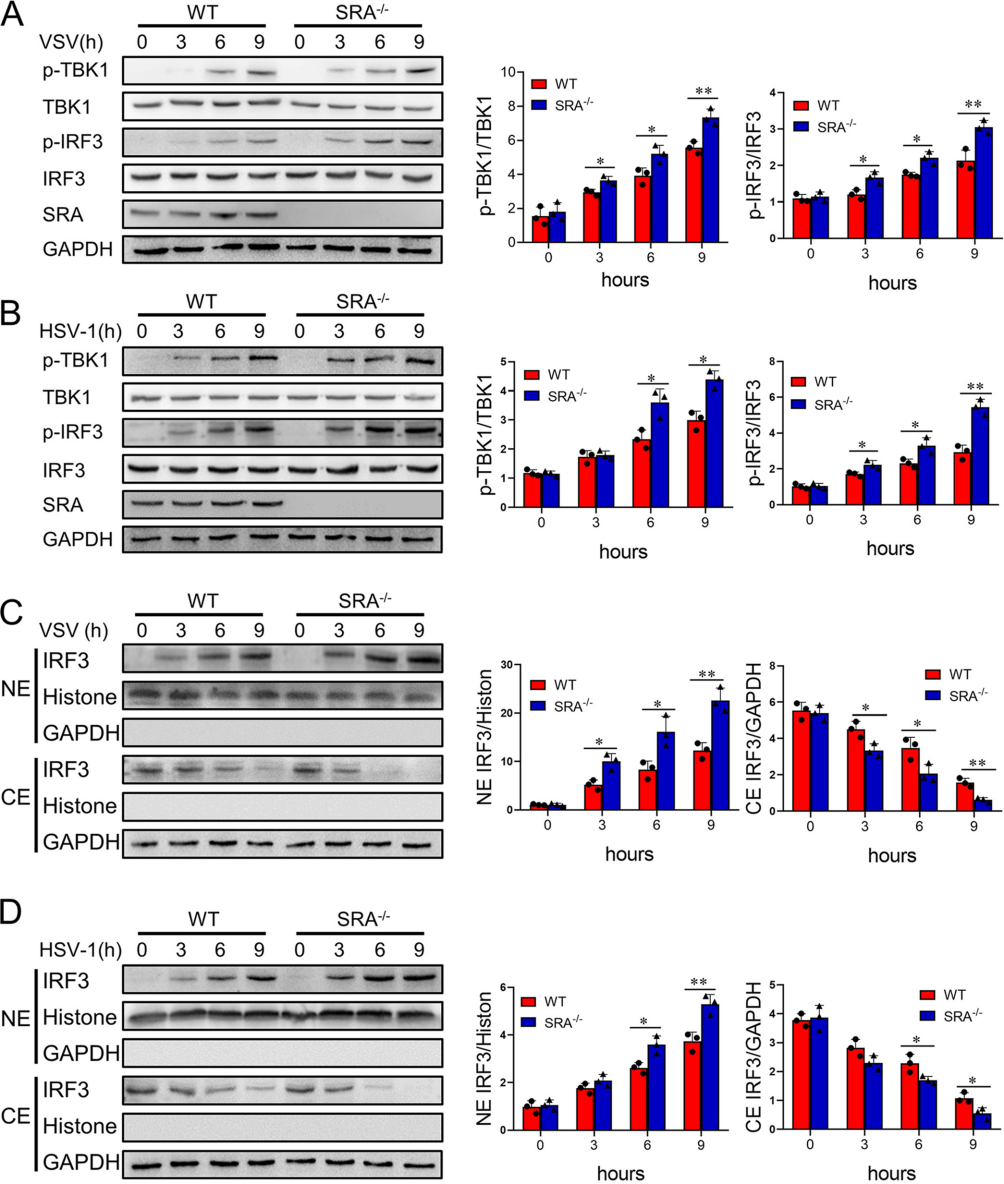
SRA negatively regulates the TBK1/IRF3 signaling pathway. BMDMs from WT and SRA^−/−^ mice were infected with VSV (MOI = 0.1) or HSV-1 (MOI = 10). (A, B) The expression of phosphorylated and total TBK1 and IRF3 of BMDMs infected with VSV (A) or HSV-1 (B) at the indicated time points. (C, D) The expression of IRF3 in the cytoplasmic (CE) and nuclear (NE) fractions of BMDMs infected with VSV (C) or HSV-1 (D). ns, not significant; *, *P* < 0.05; **, *P* < 0.01; determined by unpaired Student’s *t*-tests. Data are representative of three independent experiments with similar results.

TBK1 is a critical serine/threonine protein kinase that mediates the phosphorylation and nuclear translocation of IRF3, leading to the production of IFN-I. We found that SRA deficiency markedly enhanced the phosphorylation of TBK1 and IRF3 in VSV-stimulated macrophages (Fig. 4A). Furthermore, the absence of SRA promoted the phosphorylation of TBK1 and IRF3 in HSV-1-stimulated macrophages (Fig. 4B). Additionally, an immunoblotting assay showed that loss of SRA induced more IRF3 protein translocation to the nuclei of macrophages upon virus challenge (Fig. 4C and 4D). These results suggested that SRA is a suppressor of the TBK1/IRF3 signaling cascade for the IFN-I production in macrophages.

### SRA directly targets TBK1.

**FIG 5:**
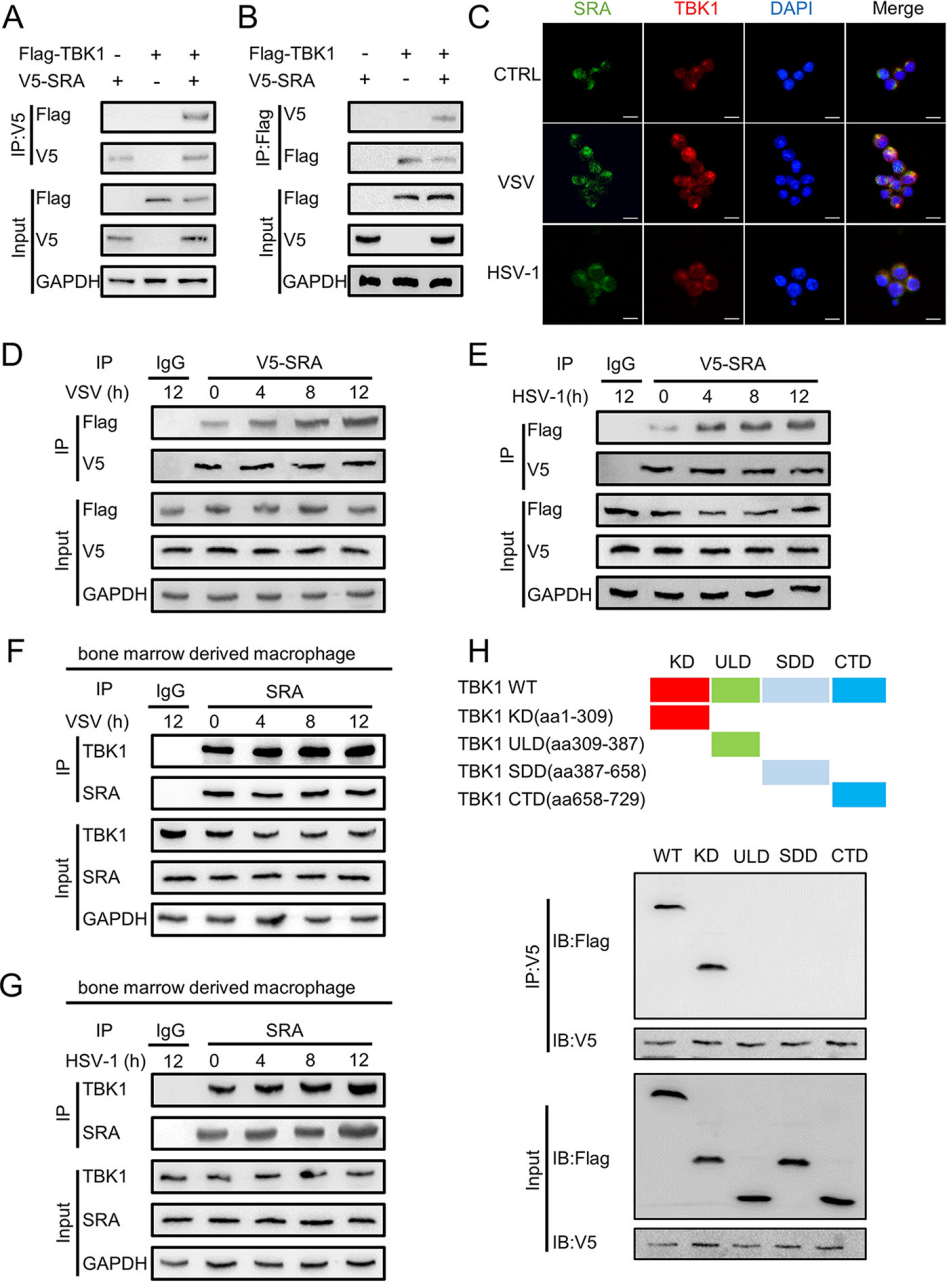
SRA directly targets TBK1. HEK293T cells were transfected with expression plasmids of Flag-TBK1 or V5-SRA for 48 h. (A, B) The association of V5-SRA with Flag-TBK1 was determined by immunoprecipitation. (C) Representative confocal microscopy photographs of TBK1 (red) and SRA (green) in RAW264.7 cells infected with VSV (MOI = 0.1) or HSV-1 (MOI = 10) for 8 h. (D, E) The association of SRA with TBK1 in macrophages infected with VSV (MOI = 0.1) (D) or HSV-1 (MOI = 10) (E) for 24 h was determined by immunoprecipitation. (F, G) The association of SRA with TBK1 was determined in BMDMs infected with VSV (MOI = 0.1) (F) or HSV-1 (MOI = 10) (G) for 24 h by immunoprecipitation. (H) The association of V5-SRA with truncation mutants of Flag-TBK1 was determined by immunoprecipitation. The top panel shows TBK1 and its truncation mutants. Scale bars = 20 μm. Data are representative of three independent experiments with similar results.

Given that TBK1 is essential for the induction of IFN-I, we sought to determine whether SRA regulates IFN-I signaling through interaction with TBK1. We determined that V5-tagged SRA interacts with Flag-tagged TBK1 in HEK293T cells (Fig. 5A and 5B). Additionally, the confocal assay validated the physical association of SRA with TBK1 in macrophages (Fig. 5C). Notably, the binding of SRA to TBK1 in HEK293T cells increased after VSV (Fig. 5D) or HSV-1 (Fig. 5E) infection. We further confirmed the association of SRA and TBK1 in mouse macrophages using reciprocal immunoprecipitation assays. Virus stimulation of the mouse macrophages similarly induced the recruitment of TBK1 to SRA (Fig. 5F and 5G).

TBK1 contains the following four domains: N-terminal kinase domain (KD), ubiquitin-like domain (ULD), scaffold dimerization domain (SDD), and C-terminal domain (CTD). To identify the domain of TBK1 binding to SRA, we generated the following four TBK1 domains, respectively, to identify its binding domain for SRA: TBK1-KD, TBK1-SDD, TBK1-ULD, and TBK1-CTD domains. The TBK1-KD domain interacted with SRA directly (Fig. 5H). These results suggest that SRA can modulate IFN-I response by targeting TBK1.

### SRA inhibits the K63-linked ubiquitination of TBK1.

**FIG 6:**
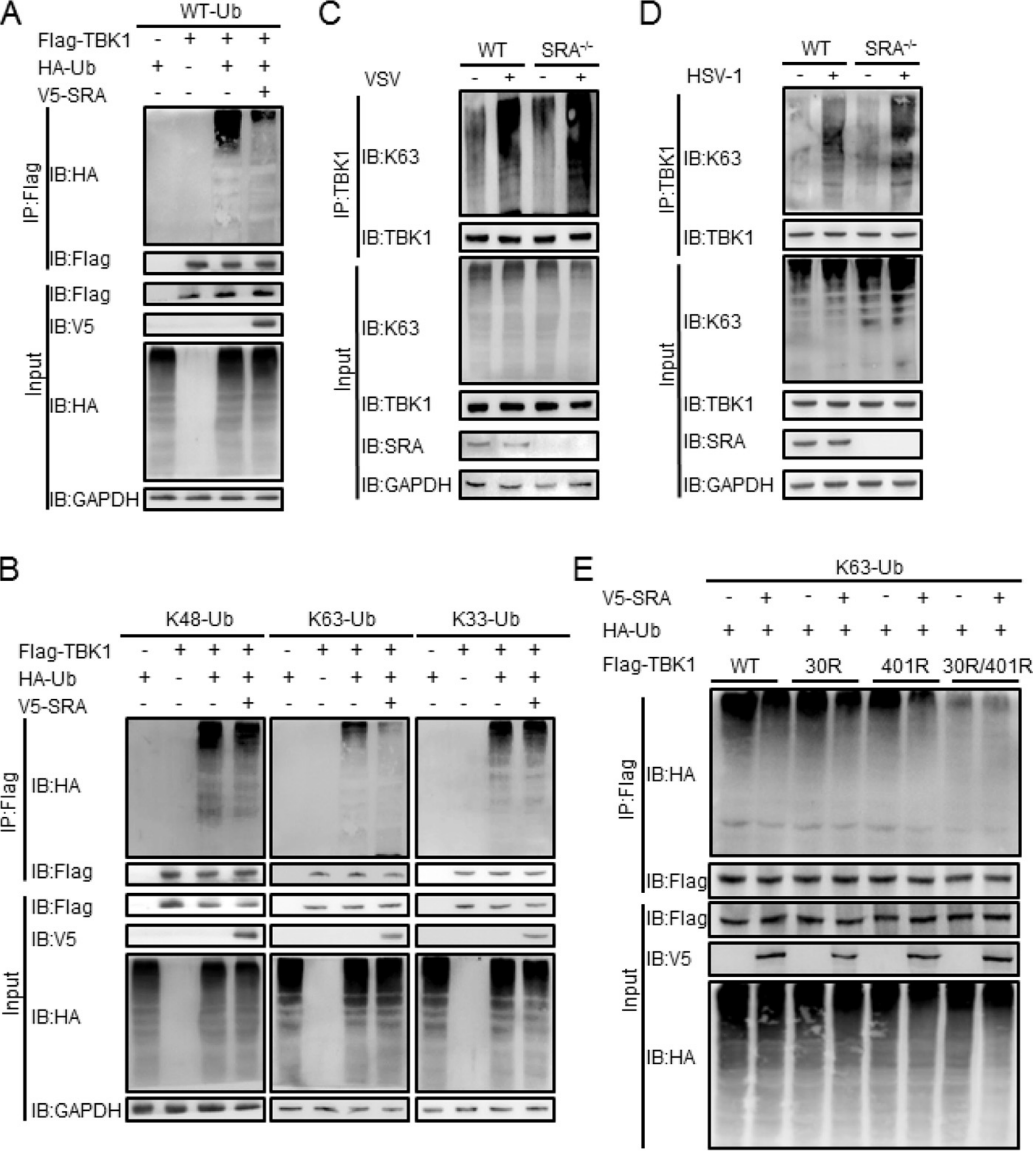
SRA inhibits the K63-linked ubiquitination of TBK1. HEK293T cells were transfected with expression plasmids of HA-Ub, Flag-TBK1, or V5-SRA for 48 h. (A) The ubiquitination of TBK1 was determined by immunoprecipitation. (B) The ubiquitination of TBK1 was determined by immunoprecipitation, followed by the cells transfected with Flag-TBK1 and V5-SRA combined with HA-ubiquitin-K48, HA-ubiquitin-K63, or HA-ubiquitin-K33 plasmids for 48 h. (C, D) The K63 ubiquitination of TBK1 in BMDMs infected with VSV (C) or HSV-1 (D) was determined by immunoprecipitation. (E) The ubiquitination of TBK1 in HEK293T cells cotransfected with expression plasmids of Flag-TBK1-WT or Flag-TBK1-K30R or Flag-TBK1-K401R or Flag-TBK1-K30R/401R along with V5-SRA and/or HA-ubiquitin-K63 plasmids. Data are representative of three independent experiments with similar results.

Ubiquitination is a necessary posttranslational modification for regulating TBK1 activity and consequently the innate antiviral immune response. To investigate whether SRA is involved in TBK1 ubiquitination, we transfected Flag-TBK1 into HEK293T cells with or without the V5-SRA plasmid. The coimmunoprecipitation experiment showed that SRA overexpression markedly decreased TBK1 ubiquitination in HEK293T cells (Fig. 6A). Notably, SRA reduced K63-linked but not K48- or K33-linked ubiquitination of TBK1, indicating that SRA inhibits K63-linked ubiquitination of TBK1 (Fig. 6B). Next, we measured TBK1 K63 ubiquitination in WT and SRA^−/−^ macrophages after VSV (Fig. 6C) or HSV-1 (Fig. 6D) infection. We observed an enhanced K63-linked TBK1 ubiquitination in SRA^−/−^ macrophages compared with WT macrophages.

Structural analysis has shown that K63-linked polyubiquitination of TBK1 at K30 and K401 is required for its catalytic activation (19, 22). Therefore, Flag-TBK1 variants with the point mutations K30R, K401R, or K30R/401R (i.e., Flag-TBK1-K30R, Flag-TBK1-K401R, and Flag-TBK1-K30R/401R) were constructed and transfected into HEK293T cells in the presence or absence of V5-SRA plasmid in HEK293T cells. Both Flag-TBK1-K30R and Flag-TBK1-K401R showed low K63-linked ubiquitination of TBK1 in the presence of V5-SRA. Flag-TBK1-K30R/401R with double mutations eliminated the differences of SRA in the K63-linked ubiquitination of TBK1. (Fig. 6E). These results indicated that SRA might affect the K63-linked ubiquitination of TBK1 at both K30 and K401 residues.

### SRA recruits USP15 to trigger K63-linked deubiquitination of TBK1.

**FIG 7:**
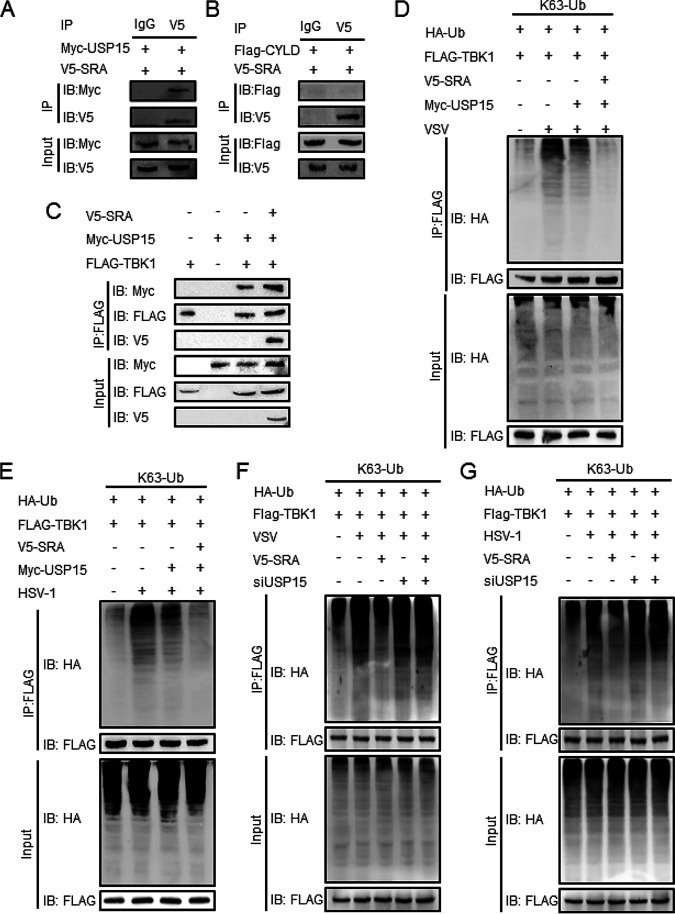
SRA recruits USP15 to trigger K63-linked deubiquitination of TBK1. HEK293T cells were transfected with Flag-TBK1 and V5-SRA along with Myc-USP15 or Flag-CYLD. (A, B) The association of V5-SRA with Myc-USP15 (A) or Flag-CYLD (B) was determined by immunoprecipitation. (C) The association of V5-SRA, Flag-TBK1, and Myc-USP15 was determined by immunoprecipitation. (D, E) HEK293T cells were transfected with HA-K63-Ub, Flag-TBK1, and V5-SRA with or without Myc-USP15. The K63-linked ubiquitination of TBK1 in HEK293T cells infected with VSV (D) or HSV-1 (E) was determined by immunoprecipitation. (F, G) HEK293T cells were transfected with HA-K63-Ub, Flag-TBK1, and V5-SRA with or without USP15 siRNA. The K63-linked ubiquitination of TBK1 in HEK293T cells infected with VSV (F) or HSV-1 (G) was determined by immunoprecipitation. Data are representative of three independent experiments with similar results.

It has been reported that the K63-linked polyubiquitination of TBK1 could be negatively regulated by some deubiquitinases, such as cylindromatosis (CYLD) (23) and ubiquitin-specific protease 15 (USP15) (12). Herein, we hypothesized that SRA might recruit some deubiquitinases to remove K63-linked polyubiquitination of TBK1. We found that ectopically expressed SRA interacted with Myc-tagged USP15 (Fig. 7A) but not Flag-CYLD (Fig. 7B) in HEK293T cells. Interestingly, overexpressed SRA promoted the interaction between TBK1 and USP15 (Fig. 7C). We also observed that SRA significantly improved the USP15-mediated deubiquitination of TBK1 in response to VSV or HSV-1 infection (Fig. 7D and 7E). Furthermore, we transfected HEK293T cells with USP15 small interfering RNA (siRNA) to examine whether USP15 is necessary for the regulatory function of SRA in the viral immune response. Western blotting showed that the K63 ubiquitination of TBK1 was similar in control or SRA-overexpressing cells in the absence of USP15 with VSV infection (Fig. 7F) or HSV-1 infection (Fig. 7G). These data suggested that USP15 is essential for SRA to reduce K63 ubiquitination of TBK1.

## DISCUSSION

Macrophages are the primary drivers of host immune defense against virus infection. As a phagocytic receptor on macrophages, SRA recognizes a series of PAMPs and modified molecules (24). Cumulative evidence showed that SRA is involved in the pathogenesis of inflammatory diseases and that its molecular mechanism may be disconnected from its phagocytic attribute (25–27). Our study demonstrated a crucial role of SRA in the negative regulation of an innate antiviral response in macrophages. SRA deficiency significantly potentiated IFN-I production in macrophages induced by either an RNA virus or DNA virus. Furthermore, we provided the biochemical evidence that SRA is directly associated with TBK1 and limited its K63-linked polyubiquitination.

SRA is a cell surface glycoprotein preferentially expressed in immune cells of the myeloid lineage, including DCs and macrophages. We previously observed that SRA expression increased dramatically on multiple subsets of MNCs in the livers of mice infected with mouse hepatitis virus (27). Based on our present data showing that SRA expression was upregulated in mice upon VSV or HSV-1 infection, we speculate that SRA upregulation might be a common phenomenon in hosts upon viral infection. Of note, DCs and macrophages are important IFN-producing cells in innate immunity. We demonstrated that SRA impaired the production of IFN-I during the antiviral response, indicating the negative regulatory loop of IFN-I production mediated by SRA in innate immune cells upon viral infection. Indeed, viruses have evolved mechanisms to escape from host immunity by inhibiting the production of IFN-I. For example, VSV infection enhanced the expression of Siglec-G to promote RIG-I degradation and inhibit IFN-I production (28). Further study must evaluate whether SRA inhibition can suppress the virus infection by modulating the IFN-I production in clinical patients.

The host defense against the invading virus recognized by PRRs rapidly induces an innate immune response and produces large amounts of IFN-I by activating IRFs, exerting a potent antiviral effect (29, 30). As a phosphokinase necessary for the activation of IRFs, TBK1 is essential in producing IFN-I and has a potent antiviral effect (29, 30). Once the viral nucleic acid or its intermediate metabolites bind to the PRR, they can recruit the corresponding junctional proteins, such as MAVS, STING, and TRIF. Subsequently, they all activate the downstream protein kinase TBK1, promoting IRF3 activation and IFN-I synthesis (31). Novel candidates targeting TBK1 have modulated antiviral-signaling pathways (8, 12). Our findings suggest that SRA interacts with TBK1, thereby regulating the IFN-I production response to DNA and RNA virus infection. Alternatively, the increased binding of SRA to TBK1 in response to DNA and RNA virus infection may represent a negative feedback mechanism regulating antiviral immunity. It is noteworthy that TBK1 is a multifunctional protein with a KD domain, followed by ULD, SDD, and CTD domains (22). The intact KD domain is responsible for mediating the downstream signaling pathway. Hu et al. demonstrated that TBK1 isoforms lack a partial KD domain and are negative modulators of the IFN-I signaling pathway (32). Indeed, several regulators could interact directly with the KD domain of TBK1, thus inhibiting the activation of TBK1 (27, 33–35). Our work proved that SRA could interact with the KD domain of TBK1 and decrease its phosphorylation. These results suggest that SRA may function as an adaptor molecule that influences the recruitment of TBK1 with their signaling adaptors, controlling the activation of the antiviral-signaling pathway.

Ubiquitination and deubiquitination are important posttranslational regulatory mechanisms in the modulation of virus-induced IFN response (36). Ubiquitin usually binds to TBK1, such as K48-, K63-, and K33-linked chains, depending on the specific environment (8–10), and all of these polyubiquitinations of TBK1 are reversible. Commonly, the polyubiquitination of K48-linked TBK1 promotes its degradation through the ubiquitin-proteasome way, while the polyubiquitination of K33- and K63-linked TBK1 enhances its activity (37). Our results demonstrated that deficiency of SRA promoted polyubiquitination of K63 linked in TBK1, and TBK1 showed low K63-linked ubiquitination in the presence of SRA. Thus, SRA decreased the K63 ubiquitination of TBK1. It is believed that the transphosphorylation of TBK1 requires a preliminary modification of coupled K63 polyubiquitin chain at Lys30 (KD) and Lys401 (SDD). It was found that single-site mutations (K30R or K401R) did not affect the synthesis of IFN, while TBK1 mutated at both sites failed to induce the synthesis of IFN, confirming that K63 ubiquitination is an essential step in IFN synthesis (22). Here, we found that SRA failed to reduce the K63 ubiquitination of TBK1-30R and TBK1-401R mutants as efficiently as that of WT TBK1, suggesting that SRA may affect TBK1 ubiquitination at Lys30 and Lys401, thereby decreasing TBK1 activity. Our results indicated that SRA might affect the conjugation of K63-linked ubiquitin chains of TBK1 to suppress TBK1 ubiquitination at both Lys30 and Lys401. TBK1 activity is regulated by E3 ubiquitin ligases and DUBs (8, 12). Our results showed that SRA binds to USP15 to decrease the K63-linked polyubiquitination of TBK1 at both Lys30 and Lys401 instead of directly regulating TBK1, which was partially consistent with a recent study showing that ubiquitin-conjugating enzyme 2S (UBE2S) recruited USP15 to inhibit the K63-linked ubiquitination of TBK1 at both Lys30 and Lys401 (12). Of note, our previous results indicated that SRA decreased K63 deubiquitination of tumor necrosis factor receptor-associated factors 3 (TRAF3) and TRAF6 (16, 19). Therefore, we propose that SRA may serve as an adaptor protein by recruiting deubiquitinases, ubiquitin E3 ligases, or other effectors to target its substrates, where they form a complex to exert their functions.

In summary, our work reveals a critical feature of SRA in controlling the cellular antiviral response against RNA and DNA viral infection, providing new insight into understanding the novel function of SRA in innate antiviral immunity.

## MATERIALS AND METHODS

### Mice.

The SRA^−/−^ mice were acquired from the Jackson Laboratory (Bar Harbor, ME, USA). WT C57BL/6J mice were obtained from the Animal Institute of Southern Medical University (Guangzhou, China). All of the animal experiments were approved by the Welfare and Ethical Committee for Experimental Animal Care of Southern Medical University.

### Antibodies and reagents.

Antibodies against SRA (sc-166184) and Ub (sc-8017) were purchased from Santa Cruz Biotechnology (Santa Cruz, CA, USA). Anti-HA (T0050) was from Affinity Biosciences (Changzhou, Jiangsu, China). The anti-Flag antibody (HT201) was obtained from TransGen Bio-tech (Beijing, China). The anti-V5 (66007-1-Ig), anti-Myc (66004-1-Ig), and anti-β-actin (20536-1-AP) were purchased from Proteintech (Rosemont, IL, USA). Antibodies obtained from Cell Signaling Technology (Danvers, MA, USA) included the antibodies against phosphor-TBK1(5483), TBK1(38066), phospho-IRF3(29047), and IRF3(4302). K63-linkage-specific polyubiquitin (5621) was purchased from Sigma-Aldrich. A human IFN-β enzyme-linked immunosorbent assay (ELISA) kit (JL19215) was purchased from Jianglai Biotechnology (Shanghai, China). Percoll (P1644) was purchased from Sigma-Aldrich (St. Louis, MO, USA). USP15 siRNA was synthesized from RiboBio (Guangzhou, China).

### Cell culture.

BMDMs were collected after 7 days of bone marrow cells cultured in Dulbecco modified Eagle medium (DMEM) supplemented with 10% fetal bovine serum (FBS) and 30% conditioned medium derived from the culture of L929 cells producing macrophage colony-stimulating factor. In addition, HEK293T cells and RAW264.7 cells were grown in DMEM supplemented with 10% FBS in a humidified 5% CO_2_ atmosphere.

### Virus infection.

For *in vitro* studies, BMDMs were cultured in a 6-well plate and infected with VSV (MOI = 0.1) or HSV-1 (MOI = 10) at the indicated time. For *in vivo* studies, mice were infected with VSV (2.5 × 10^7^ PFU/mouse) via a tail vein or HSV-1 (2 × 10^6^ PFU/eye) by eye inoculation.

### Virus titer detection.

The 50% tissue culture infective dose (TCID_50_) assay was estimated as described before (12). Concisely, Vero cells plated in a 96-well plate were infected with 0.1 mL/well of 10-fold serially diluted supernatants in quintuplicate. After incubating at 37°C for 90 min, the unattached virus was removed, and DMEM supplemented with 2% FBS was added to the Vero cells. Five days postinfection, the TCID_50_ was determined by the Reed-Muench method.

### Flow cytometry.

The expression level of SRA on macrophages in the lung, liver, and brain was assayed with an LSRII/Fortessa flow cytometer (BD Biosciences, Heidelberg, Germany). Briefly, MNCs were prepared as previously described (27). First, cells were blocked by CD16/32 (14-0161-82; Invitrogen, Waltham, MA, USA) and then stained with 7-AAD viability staining solution (eBioscience, San Diego, CA), Pacific Blue anti-mouse/human CD11b Ab (eBioscience, San Diego, CA), PE-conjugated anti-F4/80 Ab (eBioscience, San Diego, CA), anti-mouse SRA antibody (sc-166184; Santa Cruz Biotechnology, Santa Cruz, CA, USA), and the secondary antibody goat anti-mouse IgG H&L (Alexa Fluor 488) (ab150113). Flow cytometric data were then analyzed using FlowJo software (Tree Star, Ashland, OR, USA).

### Histological and immunohistochemical staining.

Paraffin-embedded lung, liver, and brain tissue blocks were cut into 5-μm slices and climbed onto poly lysine-charged glass slides. Tissue injury was determined by hematoxylin and eosin (H&E) staining. Antigen recovery was executed in a citrate buffer (pH 6.0) at 120°C for 20 min, and endogenous peroxidase activity was blocked by exposing to 3% H_2_O_2_ for 15 min. The sections were incubated with primary antibodies at 4°C overnight. Immunoreaction was inspected by the corresponding horseradish peroxidase (HRP)-conjugated secondary antibody and imaged using a diaminobenzidine kit (Beyotime Biotechnology, Shanghai, China).

### Plasmids and transfection.

The mammalian expression plasmids Flag-tagged TBK1 and V5-tagged SRA were stored in our laboratory. Flag-CYLD, Flag-TBK1 KD, ULD, SDD, CTD, 30R, 401R, and 30R/401R were obtained from BGI (BGI group, Shenzhen, China). HA-Ub, HA-Ub-K48, HA-Ub-K63, and HA-Ub-K33 were purchased from Addgene (http://www.addgene.org). The HEK293T cells were cultured in DMEM high glucose supplemented with 10% FBS. Cells were seeded in 10-cm dishes and were transfected with 10 μg plasmid with PEI (Polysciences, Warrington, PA, USA) according to the manufacturer’s instruction.

### Immunoprecipitation and immunoblotting.

Total protein was extracted using radioimmunoprecipitation assay (RIPA) lysis buffer containing protease inhibitors (Beyotime Biotechnology, Shanghai, China). Protein samples were separated on polyacrylamide gels and then transferred onto polyvinylidene fluoride (PVDF) membranes (Millipore, Billerica, MA, USA). Bovine serum albumin (BSA) (5%) was used to block nonspecific sites of the membranes for at least 1 h at room temperature. Subsequently, the membranes were incubated overnight at 4°C with primary antibodies, followed by incubation with the horseradish peroxidase-conjugated secondary antibody for 1 h at room temperature. Finally, the membranes were washed three times, and detection of the target protein was conducted with enhanced chemiluminescence (Thermo Fisher, Carlsbad, CA, USA). The densities of protein blots were quantified by using ImageJ software (NIH, Bethesda, MD).

For immunoprecipitation, the cell lysates were incubated with the indicated antibody and protein A/G PLUS-agarose (sc-2003; Santa Cruz Biotechnology) at 4°C overnight. Eluted immunoprecipitates were resolved on SDS-PAGE and examined for association of proteins of interest using specific antibodies.

### Isolation of RNA and qRT-PCR analysis.

Total RNA was extracted using TRIzol (ET101-01;Transgen, Beijing, China) and then transcribed into cDNA using the reverse transcription kit (TaKaRa, Dalian, China) as instructed by the manufacturer. SYBR green quantitative real-time PCR (qRT-PCR) was performed to determine the gene expression level using a 7900HT fast real-time PCR system (Applied Biosystems, San Francisco, CA, USA) according to the protocol provided with the SYBR Premix EX *Taq* (TaKaRa, Dalian, China). The levels of the target gene were normalized with respect to glyceraldehyde-3-phosphate dehydrogenase (GAPDH) gene expression. For primers used for the quantitative real-time PCR in this study, see Table 1.

**TABLE 1:** Primers used for the quantitative real-time PCR in this study

Primer	Forward sequence (5′–3′)	Reverse sequence (5′–3′)
VSV	ACGGCGTACTTCCAGATGG	CTCGGTTCAAGATCCAGGT
HSV-1	TGGGACACATGCCTTCTTGG	ACCCTTAGTCAGACTCTGTTACTTACCC
ifna4	TGATGAGCTACTACTGGTCAGC	GATCTCTTAGCACAAGGATGGC
ifnb1	GATCTCTTAGCACAAGGATGGC	GGCAGTGTAACTCTTCTGCAT
isg56	CAGAAGCACACATTGAAGAA	TGTAAGTAGCCAGAGGAAGG
isg15	CAACAATCTCCACTTTGCCACTG	TGGAAAGGGTAAGACCGTCCT
gapdh	AAATGGTGAAGGTCGGTGTGAAC	CAACAATCTCCACTTTGCCACTG

### Enzyme-linked immunosorbent assay.

The supernatants were collected from BMDMs or peripheral blood in the indicated experiments. The concentration of IFN-β was detected with ELISA kits according to the manufacturer’s protocols (R&D Systems).

### Confocal microscopy.

Macrophages were plated on poly-d-lysine-coated glass coverslips and then infected with VSV or HSV-1 for 1 h. Next, cells were fixed with 2% paraformaldehyde, permeabilized with 0.5% Triton X-100 in PBS for 6 min, and stained with primary antibodies against TBK1 and SRA. The primary antibodies were detected with Alexa Fluor 568-conjugated donkey anti-rabbit IgG or Alexa Fluor 488-conjugated donkey anti-mouse IgG. All of the images were acquired with a ×20 objective on an Olympus IX81 FV1000 laser scanning confocal microscope (Shinjuku, Tokyo, Japan).

### Statistical analysis.

All of the results were expressed as mean ± standard deviation (SD). Statistical significance between two groups was evaluated using Student’s *t*-tests, while comparisons of multiple groups were assessed by two-way analysis of variance (ANOVA), followed by the Student-Newman-Keul test. A *P* value of <0.05 was considered to be significant.
